# Development and clinical implementation of an LC-HRMS method for ivacaftor, lumacaftor, tezacaftor and elexacaftor in human plasma and breast milk

**DOI:** 10.1007/s00216-024-05496-2

**Published:** 2024-08-28

**Authors:** Anna B. Hansson, Hjalmar Wadström, Erik Eliasson, Mahasin Al Shakirchi, Isabelle de Monestrol, Victoria Barclay

**Affiliations:** 1 Medical Unit of Clinical Pharmacology, Karolinska University Hospital, Stockholm, Sweden; 2 Department of Medicine Solna, Division of Clinical Epidemiology, Karolinska Institutet, Solna, Sweden; 3 Department of Laboratory Medicine, Division of Clinical Pharmacology, Karolinska Institutet, Huddinge, Sweden; 4 Stockholm CF Centre, Karolinska University Hospital, Stockholm, Sweden; 5 Department of Clinical Science, Intervention and Technology, Division of Pediatrics, Karolinska Institutet, Huddinge, Sweden

**Keywords:** CFTR modulators, Therapeutic drug monitoring, High-resolution mass spectrometry, Breast milk, Human plasma

## Abstract

The four cystic fibrosis (CF) transmembrane conductance regulator (CFTR) modulators, ivacaftor, lumacaftor, tezacaftor, and elexacaftor, have revolutionised the treatment of CF by direct action on the protein target behind the disease’s development. The aim was to develop and validate a quantification method for these CFTR modulators in plasma and breast milk to better understand inter-patient variability in pharmacokinetics and treatment outcome, including the risk of adverse drug reactions. The ability to monitor CFTR modulators in breast milk enables the estimation of the exposure of breastfed infant, with a potential concern for CFTR modulator-induced liver injury. The analysis was performed on a Thermo Vanquish Flex Binary UHPLC system coupled to a high-resolution mass spectrometer (HRMS), Thermo Q Exactive. The analytes were detected using positive electrospray ionisation in full scan mode. After sample preparation by protein precipitation, the supernatant was injected onto the LC system and the analytes were separated using a Zorbax SB-C18 Rapid Res HPLC column (3.5 µm, 4.6 × 75 mm). This is the first published method for CFTR modulators in breast milk. The validated quantification range for ivacaftor is 0.0050–10 µg/mL with a coefficient of variation < 6% and a mean accuracy of 97–106%; for lumacaftor, tezacaftor, and elexacaftor, the validated quantification range is 0.050–100 µg/mL with a coefficient of variation < 8% and a mean accuracy 93–106%. A simple and sensitive quantification method for CFTR modulators has been developed and used for routine analysis of human plasma and breast milk samples since 2022.

**Graphical Abstract:**

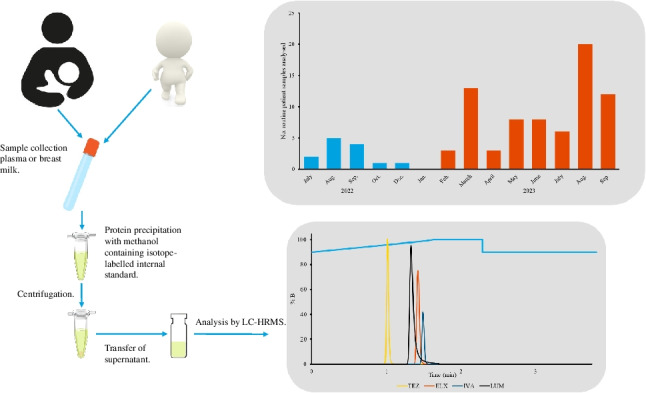

## Introduction

**Fig. 1:**
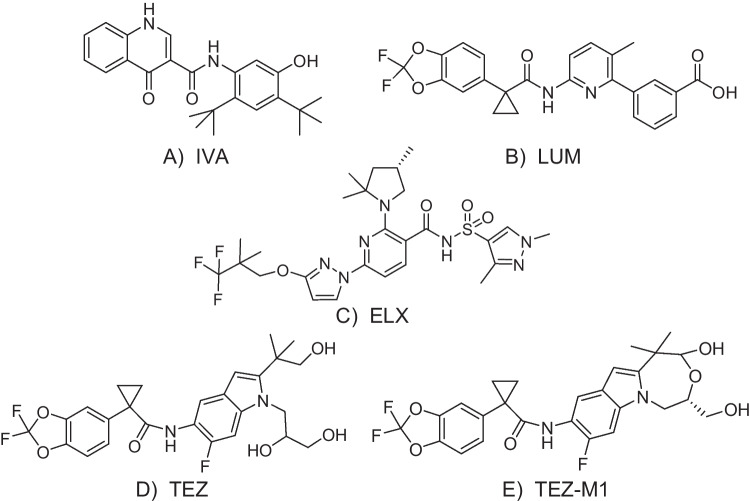
Molecular structures of the CFTR modulators, (**A**) ivacaftor, (**B**) lumacaftor, (**C**) elexacaftor, (**D**) tezacaftor, and (**E**) and the metabolite tezacaftor-M1

Cystic fibrosis (CF) is an autosomal recessive disorder caused by mutations in the gene encoding for the cystic fibrosis transmembrane conductance regulator (CFTR) protein. These mutations cause dysregulation of ion transport (Cl^−^ and HCO_3_^−^) in organs such as the lungs, pancreas, liver, and intestine. In the lungs, altered mucus characteristics lead to impaired mucus clearance, chronic infections, and inflammation, which in turn causes progressive decline in lung function [1]. Conventional CF treatments have mostly been restricted to symptomatic and supportive care. However, a novel group of active small molecule compounds, CFTR modulators (Fig. 1), improve the CFTR ion channel function in patients with CF through different mechanisms. CFTR potentiators, like ivacaftor (IVA), increase the probability that the protein channel is open. CFTR correctors, such as lumacaftor (LUM), tezacaftor (TEZ), and elexacaftor (ELX), help correct misfolding errors such as that caused by the mutation carried by nearly 90% of patients with CF, F508del-CFTR [2]. The most recent approach is the triple combination therapy (ELX-TEZ-IVA), approved as Trikafta^®^ in 2019 in the USA, and as Kaftrio^®^ in 2020 in the European Union. With the approval of the triple combination treatment strategy in patients carrying at least one copy of the F508del mutation, a highly effective modulator therapy is available for most CF patients [2].

In addition to improvements in lung function, it has been suggested that ELX-TEZ-IVA therapy ameliorates subfertility and increases the number of pregnancies in women with CF [3]. The limited reports published so far indicate that the drugs occur at low levels in milk as well as in infant plasma during breastfeeding [4–6]. However, there are reports of congenital cataracts and transient liver enzyme elevations in breastfed infants with maternal CFTR modulator exposure [6, 7]. With the now widespread use of CFTR modulator therapy, there is a growing need to understand the exposure of these agents in the unborn and breastfed child.

IVA, TEZ, and ELX are extensively metabolised in humans by CYP3A4 and CYP3A5 to active (hydroxymethyl ivacaftor, IVA-M1, TEZ-M1 (Fig. 1.), ELX-M23) or inactive (ivacaftor carboxylic acid, IVA-M6) metabolites. Some drugs that are widely used in the care of CF patients such as azole antifungals and macrolide antibiotics inhibit CYP3A4 which can lead to increased drug exposures. The recommended dosage adjustments when co-administered with CYP3A4-inhibitors in the summary of product characteristics are often based on extrapolation from other substances and the individual effect can be hard to predict [8–10]. The absorption of IVA, LUM, and ELX is greatly increased when taken with food containing fat [8, 9, 11]. Furthermore, conditions in CF such as malabsorption as well as changes in hepatic or renal physiology could affect the exposure of CFTR modulators. Therapeutic drug monitoring (TDM) could help optimise the patients’ care and reduce the risk of adverse drug reactions and drug-drug interactions, as well as treatment failure due to underexposure. Most of the pharmacokinetic data on CFTR modulators thus far comes from the market authorization holder. However, up to 10 times lower peak concentrations of ivacaftor compared to the labelling information have been reported in observational studies, with variability based on age, sex, and treating CF centre [12, 13].

A few LC–MS/MS methods have been developed and validated for the CFTR modulators in patient plasma [14–21]. Habler et al. [14] developed an LC–MS/MS method for IVA, LUM, TEZ, and ELX and their major metabolites in human serum for TDM application in clinical use. The method is based on protein precipitation and a two-dimensional chromatography setup including an online SPE column. Schneider et al. [19] developed a method for IVA and its major metabolites and LUM in plasma and sputum. Furthermore, a method for IVA, TEZ, and ELX in plasma was recently developed and applied to clinical samples from patients under steady-state treatment with ELX-TEZ-IVA [15]. Measurements of CFTR modulators in breast milk have been reported [5–7]; however, there are no published validated LC–MS/MS methods. Thus, there is a need for a simple, accurate, and highly sensitive method including all the four CFTR modulators that can be easily implemented into the routine laboratory as well as used for pharmacokinetic research purposes to help shed light on the concentration-effect/toxicity relationship of these drugs.

Therefore, the aim of this study was to develop and validate a simple method for all four CFTR modulators, IVA, LUM, TEZ, and ELX in human plasma and breast milk with a high sensitivity on a liquid chromatography high-resolution mass spectrometer (LC-HRMS).

## Material and methods

### Chemicals

The reference materials ivacaftor (IVA ≥ 98%), lumacaftor (LUM ≥ 98%), tezacaftor (TEZ ≥ 98%), and elexacaftor (ELX ≥ 98%) were obtained from Toronto Research Chemicals (North York, Canada). Tezacaftor metabolite M1 (TEZ-M1, 98.5%) was obtained from Clearsynth (Mumbai, India). The isotope-labelled reference materials ivacaftor-d_4_ (IVA-d_4_, chemical purity 96.03%, isotopic purity 95.34%) and lumacaftor-d_4_ (LUM-d_4_, chemical purity 97.22%, isotopic purity 95.93%) were obtained from Clearsynth (Mumbai, India). Tezacaftor-d_4_ (TEZ-d_4_, chemical purity 99.69%, isotopic purity 99.2%) and elexacaftor-d_3_ (ELX-d_3_, chemical purity 99.51%, isotopic purity 99.9%) were obtained from Toronto Research Chemicals (North York, Canada).

Acetonitrile HiPerSolv Chromanorm (gradient grade, used as needle wash) was purchased from VWR international S.A.S (Rosny-sous-Bois, France). Formic acid (for LC–MS) was purchased from VWR international BVBA (Leuven, Belgium). Dimethyl sulfoxide was purchased from VWR chemicals (Solon, OH, USA). 2-Propanol ACS (reagent grade) was obtained from Avantor Performance Materials (Gliwice, Poland). Acetonitrile OPTIMA (LC/MS-grade, used as mobile phase), methanol OPTIMA (LC/MS-grade), and acetone (analytical grade) were all purchased from Thermo Fisher Scientific (Waltham, MA, USA). Milli-Q water was prepared using a Milli-Q water purification system (Merck Millipore, MA, USA). Drug-free (free from CFTR modulators) citrate-plasma was supplied from the blood donation centre Skanstull, Karolinska University Hospital (Stockholm, Sweden). Drug-free breast milk (free from CFTR modulators) was supplied from the mother’s milk centre, Södersjukhuset (Stockholm, Sweden).

### Stock solutions, calibration standards, quality controls, and internal standard

Stock solutions of the analytes were prepared in dimethyl sulfoxide to concentrations of 5 g/L for IVA, LUM, and TEZ-M1; 10 g/L for TEZ; and 2.5 g/L for ELX. Intermediate solutions were prepared by further dilution of the stock solution with dimethyl sulfoxide. These intermediate and stock solutions were used to prepare six calibration levels in plasma, concentrations given in Table 1. The stock and intermediate solutions used for the preparation of the quality control (QC) samples were prepared in the same way as for the calibrators. Two sets of control samples were prepared at the following levels: lower limit of quantification (LLOQ), low, medium, and high (QCL, QCM, and QCH, respectively) (Table 1). One set was prepared in citrate-plasma and the other in breast milk.

**Table 1:** Calibration standards (CAL1–CAL6) in plasma, QC samples (LLOQ, QCL, QCM, and QCH) in plasma and breast milk

Analyte	Calibration standards, plasma (µg/mL)						Quality controls, plasma/breast milk (µg/mL)			
	CAL1	CAL2	CAL3	CAL4	CAL5	CAL6	LLOQ	QCL	QCM	QCH
IVA	0.0050	0.050	0.10	1.0	5.0	10	0.0050	0.015	4.5	8.0
LUM	0.050	0.50	1.0	10	50	100	0.050	0.15	45	80
TEZ	0.050	0.50	1.0	10	50	100	0.050	0.15	45	80
ELX	0.050	0.50	1.0	10	50	100	0.050	0.15	45	80

The internal stock standard solutions were prepared in dimethyl sulfoxide to the concentrations of 6.5 g/L for IVA-d_4_ and LUM-d_4_, 2.5 g/L for TEZ-d_4_, and 1 g/L for ELX-d_3_. The internal working standard (further referred to as IS) was prepared in methanol to the final concentrations: 0.03 mg/L IVA-d_4_, 0.3 mg/L for LUM-d_4_ and TEZ-d_4_, and 0.15 ELX-d_3_.

Stock solutions and IS were stored at − 20 °C. The calibration standards and QC samples in plasma or breast milk were stored at − 80 °C.

### Sample preparation — plasma and breast milk

Patient samples were prepared for further analysis, by protein precipitation and the addition of isotope-labelled internal standards, as follows: 50 µL of patient sample (plasma/breast milk), calibration standards, a blank, and QC (plasma/breast milk) were mixed with 600 µL of IS solution in 1.5-mL polypropylene tubes or 96-deep well plates for 30 s at 1800 rpm. The samples were centrifuged at 2100 × g (96-deep well plate) or at 11,290 × g (polypropylene tubes). The supernatant was transferred to a new 96-deep well plate or to flat-bottom glass vials and placed in the autosampler.

### The LC-HRMS method — plasma and breast milk

After sample preparation, 5 µL of the supernatant was injected onto a Thermo Vanquish Flex Binary UHPLC system coupled to a Q Exactive hybrid quadrupole-orbitrap mass spectrometer (Thermo Fisher Scientific, Waltham, MA, USA). The chromatographic separation was performed using a Zorbax SB-C18 Rapid Res HPLC column (3.5 µm, 4.6 × 75 mm, Agilent Technologies, Santa Clara, CA, USA) and three mobile phases: mobile phase A, 0.1% formic acid in Milli-Q water; mobile phase B1, 100% methanol; and mobile phase B2, 100% acetonitrile. The gradient was programmed as follows: initial — 10/90 (A/B1), 1.5 min — 1/99 (A/B1), 1.6 min — 100 (B2), 2.25 min — 10/90 (A/B1) with a total run time of 3.75 min. The flow rate was set at 0.9 mL/min.

The HRMS was equipped with a heated electrospray ionisation (HESI)-II source operated in positive electrospray ionisation mode. The spray voltage was set at 4.0 kV, sheath gas 40 AU, auxiliary gas 5 AU, sweep gas 2 AU, capillary temperature 320 °C, vaporiser temperature 450 °C, and the S-lens RF level 60. Acquisition was performed in full scan mode, with scans performed in the interval of m/z 370–650 with a resolution of 70,000, a AGC target at 3e6, maximum scan 1, and a maximum IT at 200 ms. The exact masses, mass error of 10 ppm, for the analytes are given in Table 2.

**Table 2:** The chemical formulas and nominal masses for IVA, LUM, TEZ, TEZ-M1, ELX, and the isotope-labelled internal standards. The exact masses of the ionised molecules (m/z) were used in the HRMS method

Analyte	Chemical formula	Nominal mass (m/z)	Exact mass [M + H]^+^ (m/z)
IVA	C_24_H_28_N_2_O_3_	392.499	393.21727
IVA-d_4_	C_24_H_24_D_4_N_2_O_3_	396.523	397.24238
LUM	C_24_H_18_F_2_N_2_O_5_	452.414	453.12565
LUM-d_4_	C_24_H_14_D_4_F_2_N_2_O_5_	456.438	457.15076
TEZ	C_26_H_27_F_3_N_2_O_6_	520.505	521.18940
TEZ-d_4_	C_26_H_23_D_4_F_3_N_2_O_6_	524.530	525.21450
TEZ-M1	C_26_H_25_F_3_N_2_O_6_	518.489	519.17375
ELX	C_26_H_34_F_3_N_7_O_4_S	597.658	598.24178
ELX-d_3_	C_26_H_31_D_3_F_3_N_7_O_4_S	600.677	601.26061

The software used for instrumental control was SII for Xcalibur 1.5 and Thermo Q Exactive Tune 2.11. For acquisition and data processing, TraceFinder 5.1 (Thermo Fisher Scientific) was used.

### Method validation — plasma and breast milk

The method was validated in accordance with the European Medicines Agency (EMA) guideline on bioanalytical method validation [22] for the CFTR modulators in plasma and breast milk. The method linearity, carry-over, accuracy and precision, lower limit of quantification, dilution integrity, selectivity, stability, matrix effects, recovery, and process efficiency were evaluated in plasma. Matrix effects and recovery according to EMA [22] and process efficiency were evaluated as described by Matuszewski et al. [23]. In breast milk, a partial validation was performed including intra- and inter-day accuracy and precision, selectivity, process efficiency, and short-term stability.

The linearity of the calibration curve was evaluated on three different days, according to criteria given by EMA [22]. The back calculated concentrations of the calibration level must be within ± 15% of the nominal concentration level; for LLOQ (CAL1), the back calculated concentration should be within ± 20%. Two blank samples were analysed in these runs, one with IS and one blank without IS (precipitated with methanol).

The precision and accuracy of the method were evaluated by analysing the QC samples (*n* = 6, at each level, Table 1), on three different days. Precision was calculated as the coefficient of variation (CV) for a single run (intra-day) and between the three runs (inter-day). The CV should be within 15% for QCL, QCM, and QCH; for LLOQ, the CV should be within 20%. Intra- and inter-day accuracy was expressed as a percentage of the back calculated value to the nominal concentration and is acceptable in the range of 85–115% for QCL, QCM, and QCH; for LLOQ, the accuracy should be in the range of 80–120%.

To evaluate the carry-over, a blank plasma/breast milk sample (without IS) was injected after the highest calibration standard. The peak areas of the analyte and internal standard in the blank sample were compared with the peak areas of the LLOQ. The analyte peak area in the blank sample should be < 20% and the IS peak area should be < 5% of the peaks in the LLOQ.

Dilution integrity was evaluated by preparing a QC sample that was 1.5 times higher than the highest calibration standard. The dilution control was then diluted with blank plasma at a ratio of 1:2 and 1:4 and compared with the nominal concentration.

The stability of the analytes was assessed by placing freshly prepared QCL and QCH plasma samples (*n* = 3, at each level) at different conditions (room temperature, + 8 °C and − 20 °C) and at different time intervals (1, 3, and 7 days). Three freeze–thaw cycles were performed, at − 80 °C and − 20 °C. The samples were frozen for > 12 h and the thaw time was a maximum of 1 h at room temperature. For the assessment of the stability in breast milk, freshly prepared QCL and QCH (*n* = 3, at each level) were stored at room temperature and at + 8 °C for 24 h. The freeze and thaw stability (*n* = 4 cycles) was evaluated. The mean concentration of the stored samples should be within ± 15% from the nominal concentration.

The selectivity was assessed by analysing blank plasma/breast milk samples, with and without the addition of internal standard, eight individuals for plasma and six breast milk samples from lactating women. Any chromatographic peak with the same retention time as the analytes should have a peak area < 20% of the peak area at LLOQ and < 5% of the internal standard.

The quantitative matrix effect, recovery, and process efficiency were carried out according to EMA [22] and Matuszewski et al. [23] in three different plasma matrices (citrate-, sodium heparin-, and EDTA-plasma) and eight different individuals per matrix (24 individuals) at two concentration levels (QCL and QCH). These plasma samples were spiked to concentrations corresponding to QCL and QCH before sample preparation (referred to as set A). In set B, blank plasma samples were spiked after sample preparation but before injection to corresponding QCL and QCH concentrations. The neat solution (set C) was prepared in methanol at the QCL and QCH levels. The matrix effect, recovery, and process efficiency (PE) in citrate-, sodium heparin-, and EDTA-plasma were calculated by Eqs. 1–3, where A, B, and C are the IS compensated areas for the analytes in sets A, B, and C respectively. The CV% of the matrix effect, recovery, and process efficiency at each level and matrix should not exceed 15.

In breast milk, the process efficiency was carried out by spiking six different breast milk samples at two concentration levels (QCL and QCH) before sample preparation (referred to as set A). The neat solution (set C) was prepared in methanol at the QCL and QCH levels.

1 $$\text{Matrix effect }\left(\%\right)=\mathrm B/\mathrm C\times100$$

2 $$\text{Recovery }\left(\%\right)=\mathrm A/\mathrm B\times100$$

3 $$\text{Process efficiency }\left(\%\right)=\mathrm A/\mathrm C\times100$$

### Application of the analytical methods to patient samples — plasma and breast milk

Plasma from eight patients treated with CFTR modulators was received as anonymous aliquots from samples that were to be analysed for other analyses by the Clinical Chemistry Laboratory, Karolinska University Hospital. The aliquoted anonymised plasma samples were received from the Stockholm CF Centre, Karolinska University Hospital.

Patient samples for routine TDM analysis were collected in venous blood collection tubes with sodium heparin as an anticoagulant. The plasma was then separated, by centrifugation at 2000 × g for 5 min, and stored at − 20 °C until analysis. Breast milk samples were collected in sterile test tubes and stored at − 20 °C until analysis. The patient plasma and breast milk samples were analysed by LC-HRMS as described in the “Sample preparation — plasma and breast milk” and “The LC-HRMS method — plasma and breast milk” sections.

## Results and discussion

### Method development

Some keypoints were taken into consideration during the development of the method intended for routine analysis in a clinical TDM laboratory, such as the dose-concentration relationship of the four modulators. In adults administered with the standard dose of ELX-TEZ-IVA, steady-state trough concentrations (± SD) of 0.75 ± 0.33 µg/mL (IVA), 2.05 ± 0.81 µg/mL (TEZ), and 5.49 ± 2.65 µg/mL (ELX) are reported in the registration documents provided by the market authorization holder. Largely similar trough concentrations are reported for IVA in monotherapy [24], and for IVA-TEZ therapy [25, 26]. However, the steady-state trough concentrations of IVA are much lower when administered with the inducer LUM, even though the dose is higher. The median steady-state trough concentrations in adults administered LUM/IVA 400 mg/250 mg × 2 were 0.086 µg/mL for IVA, and 12.2 µg/mL for LUM [27].

To be able to implement the method into the routine workflow in the laboratory, the validation criteria (as given in the “Method validation — plasma and breast milk” section) should be fulfilled. In addition to this, the workflow should be compliant with the standardised workflow in the clinical TDM laboratory and be easy to perform without any complicated steps during sample preparation and analysis.

A simple yet accurate method that would fit with the restrictions of a standardised clinical workflow was developed and validated. This included choosing solvents, instruments, and columns readily available in the clinical TDM laboratory.

#### Sample preparation — plasma and breast milk

For sample preparation, an easy, standardised sample preparation procedure was developed that could be easily adapted into the laboratory’s routine workflow. Protein precipitation with methanol, containing the isotope-labelled internal standards, was utilised and validated for both plasma and breast milk samples. The small sample volume of 50 µL was preferable to facilitate sample collection from children and young adults; the small sample volume is also preferable for newborns when monitoring possible exposure from the mother during breast feeding or during pregnancy. A volume of 600 µL methanol containing the isotopic-labelled internal standard was used for protein precipitation. High sensitivity and selectivity gained by using HRMS allowed for the supernatant to be transferred to vials and injected directly onto the LC system. The sample preparation included no complicated or time-consuming steps.

#### The LC-HRMS method — plasma and breast milk

**Fig. 2:**
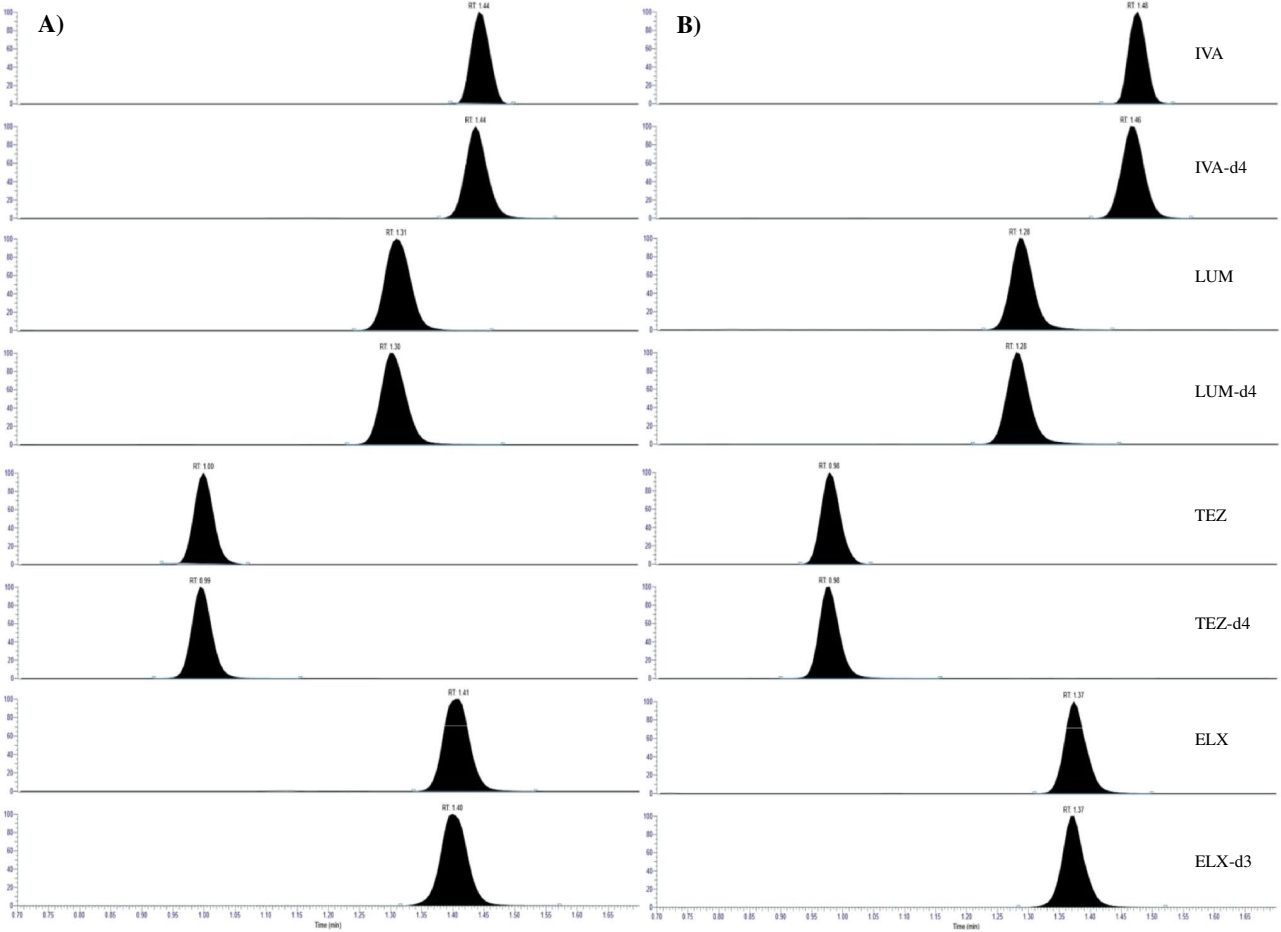
Chromatograms of IVA, LUM, TEZ, and ELX at the LLOQ level and their respective internal standards in (**A**) human plasma and (**B**) breast milk

**Fig. 3:**
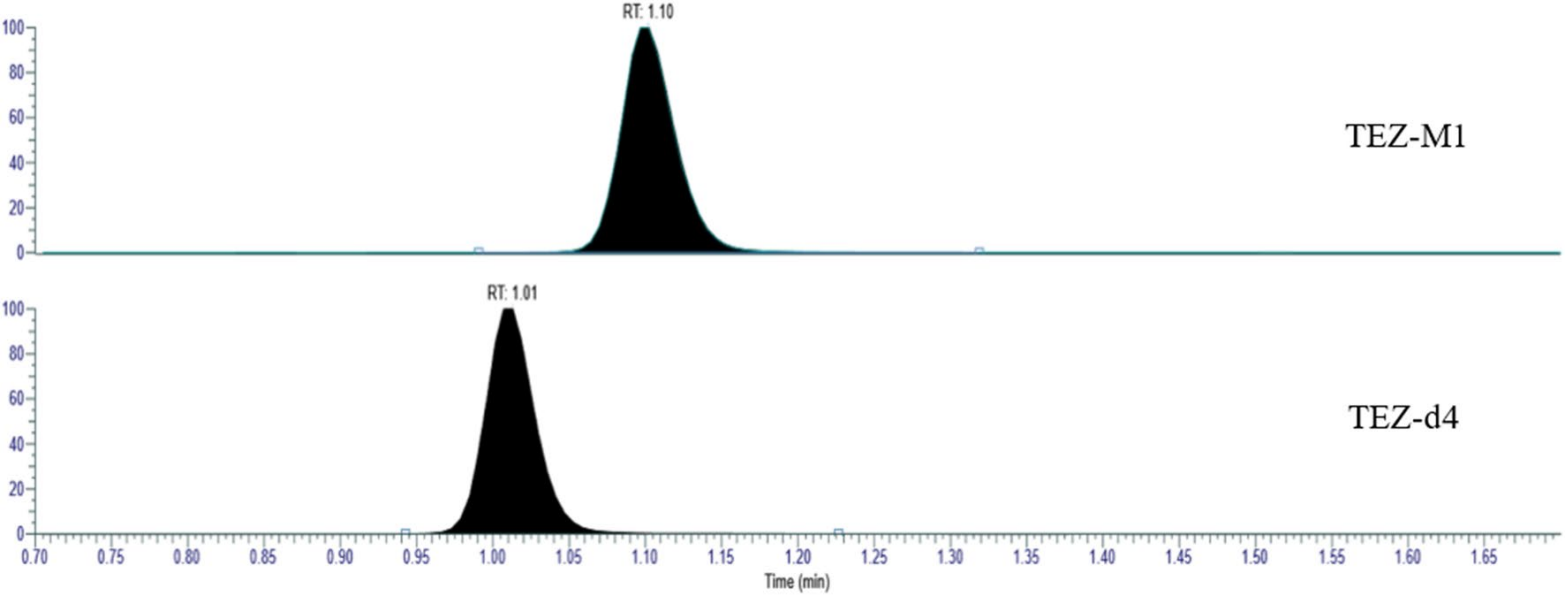
Chromatogram of the major metabolite of TEZ, TEZ-M1, and TEZ-d4 in human plasma

During the initial method validation, an increase in backpressure during the analytical run was observed when using small-particle size columns (UHPLC columns) with approximately 1.9-µm particles and an inner diameter of 2.1 mm. This was probably due to the simple sample preparation in combination with the physical–chemical properties of the analytes, i.e. highly lipophilic properties. However, instead of including a more selective, yet time-consuming, sample preparation step, a reversed-phase C18 column with particles of 3.5 µm and an inner diameter of 4.6 mm was tested. This analytical column resulted in a more stable back pressure and the separation method was further optimised for IVA, LUM, TEZ, and ELX. The four analytes as well as the metabolite TEZ-M1 were chromatographically separated (Figs. 2 and 3). However, over time, peak broadening was observed. To prevent peak broadening, a washout step with acetonitrile was included in the gradient (“The LC-HRMS method — plasma and breast milk” section).

The major metabolite of tezacaftor, TEZ-M1 (Fig. 1), was included in the LC-HRMS method development to investigate any possibility of back-conversion of TEZ-M1 to TEZ. TEZ-M1 was chromatographically separated from the analytes (Fig. 2). To investigate the risk of interference originating from TEZ-M1, the metabolite was spiked to a QC sample at the QCM level into citrate-plasma (45 µg/mL) and stored under different conditions. As the amount of TEZ-M1 decreased (monitored as the ratio TEZ-M1 to TEZ-d_4_) in the stored sample, the concentration of TEZ in the same sample did not increase, i.e. it could be hypothesised that TEZ-M1 was not back-converted to the mother substance.

### Method validation — plasma

The method for plasma was fully validated according to EMA [22]. Process effectivity, matrix effects, and recovery were assessed according to EMA [22] and Matuszewski et al. [23].

#### Linearity, accuracy and precision, carry-over, and dilution integrity

The linearity of the calibration curves expressed as the coefficient of determination (*R*^2^) was evaluated using a six-point calibration curve prepared at 3 days; linear regression was used for IVA, TEZ, and ELX; and quadratic regression was used for LUM. The weighting factor was 1/*x* and origin was excluded. The coefficient of determinations for all analyte’s calibration curves was ≥ 0.9995. The back calculated concentrations for all calibration standards were within the specifications given by EMA. The accuracy of the back calculated concentrations was within the range 94.4–107.5%.

The method showed good precision and accuracy for all analytes at all concentration levels. The intra- and inter-day accuracy for all analytes was between 92.6 and 106% at the QCL, QCM, and QCH levels. At the LLOQ level, the intra- and inter-day accuracy was in the range from 93.7 to 109.9% for all analytes. The intra- and inter-day precision expressed as the coefficient of variation (CV) was < 5% for all analytes at all levels except at LLOQ where the CV was < 8% (Table 3).

**Table 3:** The intra-day (*n* = 6) and inter-day (*n* = 18) accuracy and precision for IVA, LUM, TEZ, and ELX in plasma

Analyte	LLOQ	QCL	QCM	QCH
IVA				
Intra-day				
Accuracy (%)	106.0	98.0	104.8	102.6
Precision, CV (%)	4.2	2.0	1.5	2.6
Inter-day				
Accuracy (%)	102.9	97.3	106.2	103.0*
Precision, CV (%)	5.6	3.4	1.6	3.0*
LUM				
Intra-day				
Accuracy (%)	99.2	100.3	101.2	106.5
Precision, CV (%)	4.5	1.7	6.5	2.0
Inter-day				
Accuracy (%)	97.4	97.4	101.9	102.8*
Precision, CV (%)	3.5	2.6	3.9	3.9*
TEZ				
Intra-day				
Accuracy (%)	96.3	98.7	102.0	92.6
Precision, CV (%)	4.4	0.9	0.9	0.8
Inter-day (*n*)				
Accuracy (%)	93.7	98.5	101.7	94.2*
Precision, CV (%)	4.6	1.8	1.1	2.2*
ELX				
Intra-day				
Accuracy (%)	109.9	102.9	95.9	100.1
Precision, CV (%)	2.1	0.6	1.2	1.0
Inter-day				
Accuracy (%)	104.0	100.5	96.3	100.9
Precision, CV (%)	7.8	3.0	1.1	1.2

*n = 17

The carry-over of the method determined as the percentage of peak area in a blank compared with the peak area of LLOQ was < 1% for IVA, IVA-d_4_, LUM-d_4_, TEZ-d_4_, and ELX-d_3_; < 3% for TEZ; < 7% for LUM; and < 5% for ELX.

Furthermore, dilution integrity was assessed by diluting a high sample (15 µg/mL IVA, 150 µg/mL LUM, TEZ, and ELX) with citrate-plasma, dilution factors of 1:2 and 1:4 and five determinations per dilution. The accuracy was within 98.5–106.1 and 91.3–104.2 and CV < 2% for all analyte dilutions.

#### Selectivity, quantitative matrix effect, recovery, and process efficiency

The evaluation of the selectivity, quantitative matrix effect, recovery, and process efficiency was performed on eight individuals for each matrix (citrate-, sodium heparin-, and EDTA-plasma). All the individuals used were leftover patient samples with natural differentiation of lipidemic levels and on other pharmaceutical regimes analysed at the routine TDM laboratory. All matrices were analysed for selectivity. No interferences from other pharmaceuticals or matrix components could be observed. The quantitative matrix effect, recovery, and process efficiency were calculated using the internal standard for compensation (Table 4).

**Table 4:** The matrix effects, recoveries, and process efficiencies given as mean values (%) and CV (%) for IVA, LUM, TEZ, and ELX in citrate-, sodium heparin-, and EDTA-plasma as well as the overall results, at two concentration levels

		QCL				QCH			
		IVA	LUM	TEZ	ELX	IVA	LUM	TEZ	ELX
Citrate-plasma	Matrix effect, mean ± CV, %	111 ± 6.5	97.0 ± 6.0	112 ± 4.5	131 ± 1.4	97.1 ± 4.9	89.2 ± 3.8	94.5 ± 3.2	110 ± 1.4
	Recovery, mean ± CV, %	83.3 ± 12.2	89.8 ± 7.2	90.6 ± 6.7	75.9 ± 3.4	94.9 ± 3.0	97.4 ± 3.9	90.8 ± 4.7	75.9 ± 2.0
	Process efficiency, mean ± CV, %	91.5 ± 6.2	86.8 ± 2.3	101 ± 3.7	99.4 ± 3.7	92.1 ± 4.5	86.8 ± 4.2	85.8 ± 4.9	83.6 ± 2.1
Sodium heparin-plasma	Matrix effect, mean ± CV, %	84.7 ± 9.5	97.3 ± 3.9	119 ± 3.5	133 ± 0.6	100 ± 2.1	94.3 ± 1.5	98.4 ± 1.9	115 ± 1.5
	Recovery, mean ± CV, %	100 ± 10.9	87.3 ± 4.8	88.3 ± 4.6	75.5 ± 2.1	92.1 ± 3.2	95.1 ± 2.4	88.6 ± 3.5	74.5 ± 3.9
	Process efficiency, mean ± CV, %	71.6 ± 5.9	84.8 ± 1.5	105 ± 2.9	100 ± 2.5	92.3 ± 1.6	89.7 ± 1.6	87.1 ± 2.0	85.9 ± 4.0
EDTA-plasma	Matrix effect, mean ± CV, %	104 ± 7.7	95.8 ± 2.3	114 ± 6.5	133 ± 1.3	100 ± 2.2	90.2 ± 2.4	95.3 ± 1.9	115 ± 1.8
	Recovery, mean ± CV, %	80.1 ± 9.1	87.5 ± 4.0	89.2 ± 7.8	76.4 ± 2.8	92.9 ± 3.4	96.5 ± 3.4	92.4 ± 2.5	72.9 ± 4.7
	Process efficiency, mean ± CV, %	82.7 ± 5.7	83.9 ± 3.9	102 ± 6.5	101 ± 3.0	93.0 ± 2.3	87.0 ± 1.8	88.0 ± 2.2	83.6 ± 3.5
Overall	Matrix effect, mean ± CV, %	99.7 ± 13.5	96.7 ± 4.2	115 ± 5.4	132 ± 1.3	99.2 ± 3.1	91.2 ± 3.6	96.1 ± 2.9	113 ± 2.6
	Recovery, mean ± CV, %	82.8 ± 10.7	88.2 ± 5.5	89.4 ± 6.3	76.0 ± 2.7	93.3 ± 3.3	96.3 ± 3.3	90.6 ± 3.9	74.4 ± 3.9
	Process efficiency, mean ± CV, %	82.0 ± 11.7	85.1 ± 3.0	103 ± 4.7	100 ± 3.1	92.5 ± 3.0	87.8 ± 3.1	87.0 ± 3.3	84.4 ± 3.4

By testing the different matrices and comparing them, it was possible to make calibrators and controls in a matrix that was easy to obtain in large quantities, in our case citrate-plasma. This also allowed for the recommendation of a different matrix for the ordering physician. The matrix effects in the different matrices were seen to be consistent when compensated with IS, which concluded that calibrators and controls can be prepared in citrate-plasma and quantify patient samples in sodium heparin- or EDTA-plasma. The reason for not recommending citrate-plasma for the collection of a patient sample was due to the dilution factor of the citrate in the collection tube. The citrate buffer solution dilutes the blood and plasma to an unknown volume depending on the filling of the collection tube.

#### Stability

The stability of the analytes was evaluated in both plasma and whole blood (Table 5). In plasma, it was assessed in three different plasma matrices, citrate-, EDTA-, and sodium heparin-plasma, for short-term stability of 1, 3, and 7 days at room temperature and + 8 °C. The stability of the analytes IVA, LUM, TEZ, and ELX was tested at − 20 °C for 12 months in citrate-plasma for the ability to store calibrators and QC samples at − 20 °C instead of − 80 °C long term. The stability of the analytes in different plasma matrices and stored at different conditions was acceptable with a deviation from the nominal concentration of the samples or fresh samples within ± 15%.

**Table 5:** Stability data presented as a percentage deviation of the measured concentration versus the nominal concentration ± CV (%) (*n* = 3) for IVA, LUM, TEZ, and ELX in citrate-, sodium heparin-, and EDTA-plasma at two concentration levels. Stability in heparin whole blood patient samples (*n* = 3) for IVA, LUM, TEZ, and ELX

		QCL				QCH			
		IVA	LUM	TEZ	ELX	IVA	LUM	TEZ	ELX
Citrate-plasma	7 days room temp	3.6 ± 0.5	5.3 ± 0.2	0.5 ± 0.5	4.3 ± 0.6	4.2 ± 1.0	− 1.4 ± 2.1	− 2.2 ± 1.1	4.1 ± 0.5
	7 days + 8 °C	2.7 ± 0.7	4.4 ± 1.3	0.3 ± 1.5	4.0 ± 1.0	4.1 ± 1.2	0.7 ± 0.6	− 1.9 ± 1.4	5.1 ± 1.2
	3 freeze–thaw cycles	1.9 ± 2.8	4.1 ± 0.5	− 1.5 ± 1.0	3.1 ± 0.7	3.6 ± 1.8	− 1.8 ± 2.5	− 2.1 ± 1.5	3.0 ± 2.1
	12 months − 20 °C*	3.8 ± 3.7	8.9 ± 4.3	6.6 ± 4.8	6.0 ± 4.5	− 3.1 ± 5.4	− 0.02 ± 5.2	− 2.0 ± 5.5	− 6.3 ± 5.0
Sodium heparin-plasma	7 days room temp	− 6.6 ± 1.0	6.7 ± 1.2	1.1 ± 2.1	6.4 ± 1.0	6.7 ± 2.9	2.2 ± 1.5	0.3 ± 0.4	0.5 ± 0.3
	7 days + 8 °C	− 11.1 ± 3.2	3.6 ± 0.6	0.8 ± 0.8	5.6 ± 1.0	6.8 ± 1.4	6.8 ± 0.3	4.9 ± 0.5	− 1.1 ± 1.7
	3 freeze–thaw cycles	− 8.6 ± 7.1	7.0 ± 5.2	2.2 ± 2.6	4.8 ± 0.5	3.5 ± 1.7	1.3 ± 1.7	2.0 ± 0.8	− 0.9 ± 1.0
EDTA-plasma	7 days room temp	− 8.7 ± 3.3	4.2 ± 0.6	− 2.7 ± 1.9	1.5 ± 0.4	5.6 ± 0.9	4.8 ± 1.9	0.79 ± 1.2	2.7 ± 0.9
	7 days + 8 °C	− 8.7 ± 2.5	5.1 ± 2.3	− 2.7 ± 1.3	1.3 ± 0.7	5.1 ± 1.1	5.1 ± 3.5	− 0.19 ± 0.3	1.7 ± 0.6
	3 freeze–thaw cycles	− 11.1 ± 1.2	3.7 ± 1.7	− 2.8 ± 1.4	2.3 ± 0.8	5.3 ± 1.0	1.3 ± 0.2	0.80 ± 0.4	2.0 ± 0.4
Whole blood patient sample (6 h room temp.)	Sample 1(Trikafta®)	− 0.01 ± 0.9		0.3 ± 0.8	− 6.6 ± 1.2				
	Sample 2 (Trikafta®)	− 0.5 ± 1.6		− 0.7 ± 1.5	− 2.4 ± 2.8				
	Sample 3 (Orkambi®)	− 1.5	− 4.0						

*7.5 µg/mL IVA and 75 µg/mL LUM, TEZ, and ELX

To investigate the risk of transformation of TEZ-M1 to TEZ, the stability of the metabolite was performed by spiking citrate-plasma with only TEZ-M1 at a concentration of 45 µg/mL and stored at the same conditions as the other stability samples and then evaluated against a fresh TEZ-M1 sample. The area of TEZ-M1 decreased in the stability-tested samples but did not transform to TEZ and there was no risk of the metabolite transforming back when stored at different conditions (Table 6). To minimise the effects of variability in the analysis, the area of TEZ-M1 was compensated with TEZ-d_4_ and the area ratio was compared between a fresh control and the stability samples. The concentration of TEZ in the TEZ-M1 stability was also calculated.

**Table 6:** Stability data for TEZ-M1 (45 µg/mL) in citrate-plasma given as the deviation (%) from the initial area ratio as well as CV (%)

	TEZ-M1
1 day room temp	8.3 ± 1.9
3 days room temp	− 13.5 ± 1.4
7 days room temp	− 20.2 ± 1.8
1 day + 8 °C	5.06 ± 3.0
3 days + 8 °C	1.6 ± 7.4
7 days + 8 °C	− 6.6 ± 3.2
3 freeze–thaw cycles (− 20 °C)	− 10.2 ± 1.9

Three fully anonymised, real patient samples were used to test whole blood stability at room temperature for 6 h to simplify the preanalytical routine. Two of the patients were treated with ELX-TEZ-IVA and one with LUM-IVA. Two aliquots of the patient samples were prepared. One aliquot was centrifuged and separated directly upon arrival at the laboratory (time zero) and the other was left at room temperature for approximately 6 h before centrifugation and separation. The concentration in the 6-h sample was compared with the concentration measured in the zero sample. The analytes did not show any degradation when left at room temperature for 6 h.

### Method validation — breast milk

The method for breast milk was partially validated as described in the “Method validation — plasma and breast milk” section. The method was validated using calibration standards prepared in plasma and QC samples prepared in breast milk (Table 1).

The precision and accuracy experiments were determined using the QC samples at four concentration levels in breast milk. The precision and accuracy for the breast milk method were fulfilled according to the guideline given by the EMA [22] with an intra- and inter-day accuracy for all analytes within the range 89.9–105.3% at the QCL, QCM, and QCH levels. At the LLOQ level, the intra- and inter-day accuracy was between the range 86.0 and 118.8% for all analytes. The intra- and inter-day precision (given as CV) for all analytes was < 4.1% at all levels except at the LLOQ level where the CV was < 6.4% (Table 7).

**Table 7:** The intra-day (*n* = 6) and inter-day (*n* = 18) accuracy and precision for IVA, LUM, TEZ, and ELX in breast milk. The process efficiency (PE) (*n* = 18) and CV% for the analytes in breast milk

Analyte	LLOQ	QCL	QCM	QCH
IVA				
Intra-day				
Accuracy (%)	115.6	99.1	99.4	100.2
Precision, CV (%)	3.4	1.7	1.0	1.4
Inter-day				
Accuracy (%)	118.8*	101.7*	101.4	102.6
Precision, CV (%)	3.4	3.2	1.8	2.1
Process efficiency, mean ± CV, %		99.4 ± 8.1		97.4 ± 2.2
Stability				
24 h room temp., Dev. ± CV%		− 2.0 ± 1.4		− 1.0 ± 1.3
24 h + 8 °C, Dev. ± CV%		4.9 ± 3.3		1.1 ± 1.5
6 months − 20 °C, Dev. ± CV%		− 8.9 ± 1.9		− 4.9 ± 1.7
LUM				
Intra-day				
Accuracy (%), intra-day	96.6	101.1	100.6	102.5
Precision, CV (%)	1.8	1.1	1.1	2.3
Inter-day				
Accuracy (%), inter-day	95.3	103.4	103.7	105.3
Precision, CV (%)	1.9	3.1	3.8	4.1
Process efficiency, mean ± CV, %		89.4 ± 2.5		99.6 ± 2.4
Stability				
24 h room temp., Dev. ± CV%		− 3.2 ± 1.1		− 0.4 ± 0.1
24 h + 8 °C, Dev. ± CV%		6.9 ± 1.4		0.4 ± 0.2
6 months − 20 °C, Dev. ± CV%		− 8.5 ± 1.9		− 6.0 ± 2.6
TEZ				
Intra-day				
Accuracy (%)	86.0	92.1	93.1	92.9
Precision, CV (%)	1.9	1.0	0.9	1.1
Inter-day				
Accuracy (%)	89.9	95.0	96.4	95.8
Precision, CV (%)	6.4	3.6	2.9	2.9
Process efficiency, mean ± CV, %		87.5 ± 3.4		97.7 ± 1.0
Stability				
24 h room temp., Dev. ± CV%		− 21 ± 3.0		− 16.6 ± 1.6
24 h + 8 °C, Dev. ± CV%		− 5.7 ± 3.5		− 10.2 ± 0.5
6 months − 20 °C, Dev. ± CV%		− 8.4 ± 2.1		− 4.4 ± 1.5
ELX				
Intra-day				
Accuracy (%)	110.9	97.3	89.9	96.0
Precision, CV (%)	1.2	1.1	1.2	2.1
Inter-day				
Accuracy (%)	116.0	99.6	91.1	97.3
Precision, CV (%)	4.0	2.9	2.6	2.8
Process efficiency, mean ± CV, %		86.6 ± 3.4		95.1 ± 2.2
Stability				
24 h room temp., Dev. ± CV%		− 7.2 ± 1.9		− 9.8 ± 1.2
24 h + 8 °C, Dev. ± CV%		3.9 ± 1.4		− 1.8 ± 1.1
6 months − 20 °C, Dev. ± CV%		− 8.6 ± 1.1		− 4.9 ± 2.9

*n = 17

The process efficiency was investigated using breast milk from six different lactating women. The IS normalised process efficiency was calculated at QCL and QCH, mean PE values and CV are given in Table 7. The process efficiency ranged between 86.6 and 99.6% at both levels for all analytes and the CV for the six lots of breast milk were < 8%. It was concluded that plasma calibrators and controls can be used when quantifying the CFTR modulators in breast milk. There were no major differences in PE between plasma and breast milk matrices.

The selectivity of the method was determined by analysing the six breast milk matrices. No interfering peaks were observed at the retention times for the analytes nor at the retention times of the isotope-labelled internal standards.

The short-term stability in breast milk was evaluated at room temperature and + 8 °C. IVA, LUM, and ELX were shown to be stable at room temperature for up to 24 h; however, this criterion was not fulfilled for TEZ. The recommendation for breast milk sample storage was determined to be + 8 °C for up to 24 h. For longer storage, the samples need to be frozen at − 20 °C.

In summary, it was concluded by the validation results that breast milk samples can be accurately quantified using the same calibration standards as for the plasma method.

### Clinical implementation and application of the analytical methods to patient samples — plasma and breast milk

**Fig. 4:**
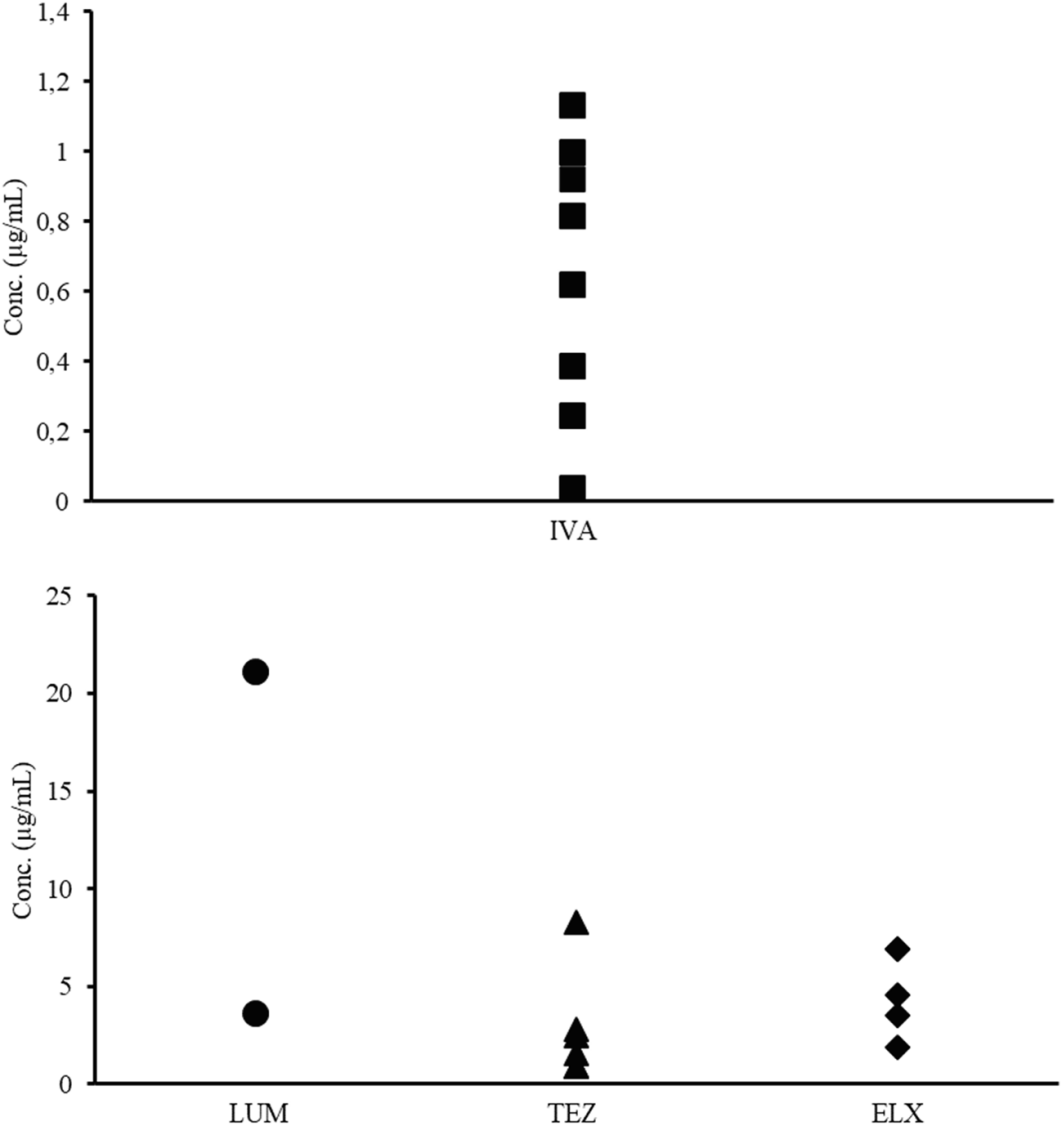
Quantified concentrations of IVA (*n* = 8), LUM (*n* = 2), TEZ (*n* = 6), and ELX (*n* = 5) during method validation

During the validation of the method, eight, fully anonymised plasma samples from patients undergoing treatment with the CFTR modulators were acquired by leftover material used in other assays. Five of the samples came from patients treated with ELX-TEZ-IVA, and one sample was from a patient treated with IVA-TEZ and two patients treated with IVA-LUM (Fig. 4).

Between July 2022 and September 2023, we have been using the method in a therapeutic drug monitoring context and approximately 80 clinical plasma samples from patients undergoing treatment with the CFTR modulators have been analysed. In addition, three breast milk samples have been analysed on mothers.

## Conclusion

A simple and sensitive LC-HRMS method for simultaneously quantifying ivacaftor, lumacaftor, tezacaftor, and elexacaftor in human plasma samples and breast milk has been validated and is used in the routine TDM analysis of CFTR modulators. During method validation, the major metabolite of tezacaftor, TEZ-M1, was only studied as a potential interference in the method. Moreover, the method was not developed for any of the metabolites to the CFTR modulators, and validation was not assessed for TEZ-M1 or the other metabolites. Yet, the method was thoroughly validated for ivacaftor, lumacaftor, tezacaftor, and elexacaftor according to the European Medicines Agency guideline on bioanalytical method validation [22] and Matuszewski et al. [23]. During the time for method validation, the EMA guidelines were the current guideline, and the validation was performed accordingly. The validated method can be used in future studies to gain a greater understanding of the concentration–response and concentration-toxicity relationships of these compounds.
